# An Essay on Bilious Fever

**Published:** 1841-09

**Authors:** Lunsford P. Yandell


### WESTERN JOURNAL

OF

### MEDICINE AND SURGERY.

---

SEPTEMBER, 1841.

---

ART. I.—*An Essay on Bilious Fever.* By LUNSFORD P. YANDELL, M. D.

Sanos sospitare agrosque sanare.

Bilious Fever is a most prevalent disease in the valley of the Mississippi. It is, also, a most fatal disease. It carries off the infant at the breast, the old man, the tender youth, the young man, and the man in the prime and vigor of useful life. It is the *Great Destroyer* in all our south-western States. The best minds of the medical profession have been devoted to its study, and to the means of subduing it, in both hemispheres, and on both sides of the line; but yet it remains unconquered. We have ascertained, pretty accurately, the circumstances under which it takes its rise, and are generally agreed upon the name of its imaginary cause; great improvements have, also, been made in the mode of treating it, and higher success crowns the efforts of modern practitioners; but, despite all, it continues often to prove fatal, and in one of its forms, is still the terror of south-western physicians.

Aetiology.—Nothing in regard to Bilious Fever is better settled, than that the poison which generates it is developed by the drying up of ponds and water courses, in a fertile region of country, under an ardent summer and autumnal sun. Wherever moisture is present to co-operate with organic matter in warm weather, there is decomposition going on, and there is found bilious disorder—especially by the bottoms of streams or ponds recently exposed to the air and the sun, does the poison of fevers seem to be eliminated. The draining of a mill-pond, if done late in summer, or in the fall, hardly ever fails to spread miasmatic diseases far and wide among the inhabitants of the neighborhood. The following is an instance, in point, which has never been published:—The proprietor of a mill on a small stream in Ohio was in the habit, every year in early summer, when the waters became low, of draining the pond, by raising the flood-gate. No injury to the health of his neighbors resulted, so long as the draining process was confined to early summer: the inhabitants of a village bordering upon the stream were not particularly subject to fevers. But during a wet summer, in 1839, as the stream continued full, he was induced to postpone draining the pond until late in August. The consequence was, that a most formidable fever broke out in the village, and prevailed to an extent before unknown in it. The owner of the mill was compelled to draw off the water, next spring, the pond having been condemned as a public nuisance, and the summer which followed was one of health in all the surrounding neighborhood.

The droughts of our summers, by exhausting the streams and exposing the deposits of organic matter on their margins and in their beds, are known to all practising physicians to be the almost certain harbingers of fever in some one of its varied forms. And in reviewing the record that we have kept of our cases of fever, we are able to trace nearly every one of them to the borders of some pond or stagnant stream. Year after year, we find that our autumnal business has been almost wholly confined to the vallies of the neighboring creeks and rivers.

In the neighborhood of Louisville, and particularly to the South, lies an extended district of flat country dotted all over with ponds; it is called the "Pond Settlement." In the spring, and during the early summer, this region is salubrious, for the ponds, as yet, are full of water. But when they begin to be exhausted in July, and their beds send up exhalations under the solstitial heats, fever, in every shape, rarely ever fails to attend. If this district ever escapes this unwelcome visitant, it is in a rainy season when the ponds are kept full. In an early day, and, in fact, until within a late period, this city was regarded as one of the most unhealthy places in the valley of the Ohio; but such has been the beneficial effects of draining and tillage, that it would now compare, in the healthfulness of its citizens, with any other river town in the West. This is particularly true of the central and densely settled parts of the city. The suburbs continue to be visited by fever. A line might be traced around the city marking the limits of the healthy atmosphere, and the point at which malaria begins to assert its authority. Within certain limits the air is pure, but beyond these, on the three sides of the town exposed to the miasms of the ponds, it is still unsafe to reside. The explanation of these facts is easy. The inhabitants in the interior are remote from the source of the poison, and are, moreover, protected by the intervening buildings; whilst those who dwell on the frontier inhale the poison emitted by the ponds that have not yet been drained. As the draining progresses the boundaries of health are found to be sensibly and rapidly extended, and the region upon which the pestilential vapors have so long rested, like a dismal incubus, promises to become, in a few years, under a judicious system of ditching and culture, one of salubrity and great value. The environs of Louisville only require to be rid of the stagnant water which remains, to be rendered as healthy as Main street, which was as often visited by malignant fevers as they now are, while large ponds exposed their filthy waters to the sun, in quarters now covered by pavements and houses.

It is interesting and instuctive to remark, how coincident are all the circumstances attending the rise and propagation of bilious fever, in countries so remote as Italy and the Valley of the Mississippi. In following the historian of the Campagna di Roma, one cannot fail to be struck with the exact identity of the causes which have desolated that remarkable region, and are traced everywhere in this country as exciting malarious diseases. The Campania, we learn from Roman history, was once a densely populated and beautiful country—the site of the thirty cities of Latium with its dependant villages. It is now nearly a wilderness—a long series of wars with their Roman neighbors laid waste its fields, depopulated its villages, and exhausted the resources and energies of the remnant of its people. As a consequence, the mountain streams, which industry had confined to narrow channels spread unchecked over the low valleys, and formed anew the lakes and pools which had long been dried up. The pestilence followed quickly on the footsteps of war, to complete the work of desolation. A poisonous atmosphere was gathered over the Campagna, and spread its baleful influence far around. Town and village gradually disappeared, until the sites of many of them could scarcely be distinguished. The progress of depopulation was at first so slow as to be almost imperceptible. "The outskirts were thinned by fevers which, returning at the return of mid-summer or of autumn, gradually compelled the inhabitants to retire towards the centre of population, or remove at once to more elevated and salubrious situations. By degrees the range of the disease extended, drawing a narrower and still narrower circle around the fated spot. Years might pass away before its hold was made secure, but its violence was redoubled as it approached the centre, until one or two seasons were sufficient to complete the work of destruction."*

But the Campagna thus desolated by malaria revived, when Rome, carrying her conquests beyond the confines of Italy, became the centre of a great empire, and wealth, with all its attendants, flowed in upon her. People revisited its deserted fields, and rebuilt the villages. Its streams were guided once more into their proper channels, and the stagnant pools ceased to send up their noisome effluvia. Luxuriant harvests covered the beds of exhausted marshes, and the villas of some of the first citizens of Rome crowned many of the heights of the region where the pestilence but a few years before had held undisputed sway. And in this prosperous state the Campania continued, so long as Rome remained the seat of empire. But with the removal of the Popes to Avignon commenced a new period of decay. The villas were abandoned to the charge of indolent agents and slaves, and fell gradually into ruins. Many of the villages were deserted, and smitten by the reigning spirit of luxury and effeminacy, and galled by an iron despotism, all lingered on in a doubtful and precarious existence, until the inroad of the barbarians completed the ruin of the country. The population of Rome itself dwindling down to seventeen thousand souls, the Campagna was deserted, except by a few miserable beings who dragged out an existence of thirty or forty years, rarely ever reaching sixty.

And thus, with the neglect of its culture, and the accumulation of water upon its surface, the Agro Romano sunk again to the lowest point of desolotion. The facts are deeply interesting to the medical philosopher. It is seldom, indeed, that we are ever able to trace more satisfactorily the connexion between the phenomena of disease and their causes. And it is not the least interesting fact in the history of this country, that, since the removal of the Papal Court to Rome again,

*New-York Review.

there has been a gradual improvement in the salubrity of the Campania—that event being followed by the return of laborers to its fields, and the draining of the Pontine marshes by Pius the 6th. That immortal enterprise, the writer from whom I have quoted remarks, had it been followed up by the draining of other portions of the Agro Romano, might long since have reclaimed it. Enough was done to show where the evil lay, and to indicate the mode of removing it.

The malaria, it is well understood, is confined to the lowlands of the Campania. High situations are exempt from it; and it would be easy, it is stated by writers who have visited it, to form a scale presenting, at different points of elevation, the gradation of noxious, bad, suspicious, passable, good, and, finally, excellent air. Monte Mario, at the gates of Rome, is healthy throughout the year. The village of St. Oreste, which stands midway on the ascent of Mount Soracte, and yet is desolated by autumnal fevers, seems to be an exception. "Here, however, the peculiar structure of the mountain itself affords an explanation of the variation. It is a solid mass of nearly naked rock, with but here and there a scanty growth of shrubs and low wood to shelter it from the exhalations of the marshes below."

The unhealthy season at Rome commences not until towards the middle of July. The shepherds remain with their flocks through nearly the whole month of June, taking the remainder of this interim of good air to gain the mountains by slow marches. By this time the smaller streams and shallow pools are dried up, and the two rivers, the Anio and the Tiber also load the air with noxious exhalations. The degree of unhealthfulness of any particular season depends in a great measure upon the autumnal rains. When these are delayed or scanty, the fever obtains a stronger hold and spreads with greater rapidity. But an early and great fall of water produces an immediate change in the atmosphere, not only cooling but purifying it, and by renewing the half-drained streams covers up the reeking sources of malaria.*

*For these details concerning the Agro Romano, I am chiefly indebted to a spirited writer in the New York Review.

I fear the reader will deem that I have devoted too much space to the history and physiognomy of a region so remote from the scene of his professional labors. All that I can say is, that the interest of the subject has appeared to me to justify the length of the details. The Campagna di Rome is not only famous for the malignity of its fevers, but as having been longer the seat of febrile diseases than any locality of which we have any authentic history. Long before the time of Cicero and Pompey, it had been desolated by the poison which constitutes the scourge of the magnificent valley of the West. And, after the lapse of more than twenty centuries, the same malaria continues to hover over its plains, and to walk in darkness through its villages and cities. The Campania has twice been rendered salubrious by a wise system of draining and tillage ; and twice has it been yielded back again to the dominion of the pestilence. Health has followed the footsteps of industry, and, with the return of barbarism or indolence, the waters have spread over the plains unchecked, and fevers have returned with all their pristine malignity.

To all this we have many analogies at home. I have cited the case of the city of Louisville, where draining and cultivation have, in a few years, introduced the most favorable changes. The clearing away of the forests, with a consequent exposure of a soil, rich in the remains of organic matter, to an ardent sun, on the other hand, has been a prolific source of febrile disease in all the south-western states; for the barrier which trees oppose to the spread of malaria has been removed from the water courses, and the noxious exhalations from water, as well as from the virgin soil turned up by the plough, have contributed to taint the air. Along all our streams disease is known to have become much more prevalent, since the shrubs and trees fringing their borders were cleared away. I remember a beautiful country seat, in one of the Western states, which lies near a small river that becomes stagnant in summer and autumn. In early times, and while the river was yet surrounded by a dense forest, the locality was one of the most healthy in all that neighborhood. A case of fever, for some years after a large family settled upon it, was of rare occurrence. But as the fields were extended and the forests removed, the visits of this malady became more frequent. The trees which once stood between the dwelling and the river have now been hewn away, and the winds, laden with the seeds of disease, meet with no obstruction. The place is subject to autumnal fevers of a malignant type. This is but one of innumerable instances that might be cited; but so familiar must the observation be to all practitioners, that it would be superfluous to multiply examples. The river to which I have just referred is quite noted for its insalubrity, and it is worthy of remark, that it is also the site of a great many mills. From the point where the first mill is erected, to the mouth of the river, hardly a healthy situation in the neighborhood of it can be found.

Another fact, connected with the propagation of miasms, of the highest importance, is their dependence for transmission upon the winds. A house built upon the eastern, or the north-eastern bank of a river or pond, receives the prevailing winds after they have swept these sources of malaria, and will be more unhealthy than one which stands in a southern or westerly direction from such collections of water. Houses so built as to expose their open doors and windows to the breezes coming over stagnant water or marshes, are more subject to fevers than those in the same village, on the same street, which oppose their rear, with closed entrances, to the wind. This is the statement of historians with regard to Rome, and it is the experience of all towns and villages where febrile diseases have been generated by collections of stagnant water. In Rome, we are assured, that the interposition of so slight a barrier to the poison, as a piece of guaze, spread over the window-openings at night, will protect the sleeping inmates; whilst those who sleep with their windows open, and without such defence, in the direction of the marshes, are almost sure to be attacked by fever. In all parts of the Western country, situations to the south of streams, especially if elevated, are comparatively healthy; those which lie as far from the water, north, being unhealthy. Situations east, and north-east, are subject to fevers; and the explanation is found in the fact, that the south, and south-west winds are prevalent in summer and autumn, with an interchange, occasionally, of a breeze from the north-west, later in the season.

If upon this branch of my subject I seem to have dwelt at undue length, my apology is, that it involves principles of the deepest importance to the community. "*Sanos sospitare, œgrosque sanare*," I have adopted as my motto, and I hold the first to be, not less than the last, the duty of the physician. The public health is a matter of such vast concern, that one may well be pardoned for a little prolixity in the detail of facts which have an intimate bearing upon it. And, if I am not greatly mistaken, I have indicated, in the history I have given of the rise and propagation of malaria, the mode by which the range of the diseases it produces may be greatly limited. Let the hygienic precepts that they suggest be properly inculcated upon the people, and they will be more industrious in removing the sources of miasmata. Let them be taught that fever is the sure penalty annexed to a residence on certain banks of water-courses, and that it may, with great certainty, be avoided by selecting a site at a distance from them, and it is not probable that they will persevere in violating so obvious and salutary a principle. It is not saying too much to assert, that, by the observance of the laws which experience and observation have established on this subject—by planting and draining, by cultivating the soil, and by the selecting of proper sites for dwellings—half the fevers might be prevented which now afflict the country, and annually rob it of many of its best citizens. I speak with the greater earnestness in reference to these things, because, while the truth of all that I have said is generally known and admitted by medical men, they do not appear to me to have exerted themselves as they might have done, to reform the people on these points of hygiene. To my mind, it involves truths of the deepest practical import, and it is only by impressing them upon individuals and the public authorities that we can hope for reform, and the improvement of the general health.

I am aware that it is contended by some, not without plausibility, that there is *no specific cause* of fever; but that it is excited by vicissitudes of atmospherical temperature, and by the chilling effects of the vapors condensed into fog by the coolness of the evening. But the fact, that fever has a specific type—as distinct and peculiar as small pox or measles—that houses, in particular situations near water, are healthy, while others, not nearer, but to the leeward, are unhealthy, both being alike enveloped in the fogs arising from the water—that in places once famed for their insalubrity health has been brought about by draining, while the air continues to be surcharged with aqueous vapor exhaled from the neighboring streams, as is strikingly true of a city to which I have already alluded*—and that the draining of a mill-pond, by which the quantity of atmospherical moisture in its vicinity is diminished, taints the summer and autumnal air in all the surrounding region—all point to a specific cause. And this cause we continue to refer to marshes, or the exhalation of moisture from a bed of putrifying organic matter; but we are compelled to admit, at the same time, that this poison has never been detected by any chemical test as a constituent in the atmosphere of infected places. Broschi directed a long series of cautious experiments to the solution of the problem; and in the air of one of the most pestilential regions in the Campagna di Roma he thought, by his first analysis, he had detected the miasmatic particles. On renewing the experiments, however, and collecting the vapor with new precautions, he was unable to discover the slightest trace of either vegetable or animal matter. Moschati examined the air of some insalubrious rice-fields in Tuscany, from which it appeared that it contained albuminous flocculi, but the nature of which he could not determine.† Prout, in his Bridgewater Treatise, mentions that, during the prevalence of cholera, in London, he found the atmosphere to be increased in weight, and stead-

*Louisville.

†Records of General Science, quoted by the American Jour. of Med. Sci. for Nov. 1836.

ily so, and the explanation of the fact he supposes to be, that some gaseous body was diffused through the air of the city, considerably heavier than the air it displaced; but the character, and even the existence, of this gas, was a matter of conjecture. M. Rigand de l'Isle made a series of experiments in the marshes of Languedoc with a view of detecting the poison of fever. He condensed the dew upon glass, and, like Moschati, found flocculi possessing the properties of animal matter, but without being able to prove that he discovered the miasm.* A more laborious series of experiments were instituted by Boussingault, with the same view, and although he detected organic matter in the vapor over the marshes, he was not more successful than the preceding chemists in insulating the peculiar poison. I will only add, that such has been the result of other investigations, instituted with a view to the discovery of this most prevalent but hidden poison, which, as we have seen, is as rife and as potent now, in the marshes around Rome, as it was in the time of Tacitus, and which all that has hitherto been done by the hand of industry in our south-western valley has not materially abated—I ought, perhaps, rather to say, that the amount of cultivation in the West has only fully developed the poison, which a higher cultivation may subdue; and with this remark, I dismiss the first branch of my subject.

### PATHOLOGY OF FEVER.

This is one of the vexed questions of the day. It is not my intention to present a history of all the theories of fever which have been proposed in the various ages of Medicine, much less to attempt an estimate of their several merits. This I should deem a most unprofitable consumption of time; and I shall therefore confine my remarks, on this head, to the two most prominent of the modern theories, the anatomical theory of Broussais, Louis, Bouillaud, Bretonneau, &c., and the

*Ib. loc. citat.

congestive theory of Armstrong, Johnson, and others, presenting afterwards, a view of the phenomena of fever as they appear to be successively developed. I shall speak first of the congestive theory.

According to Armstrong, the essential or proximate cause of fever consists in a congestion of the internal veins of the body, and particularly those of the portal circle. It was a belief expressed in his earlier writings, that, in the pathology of fever, the right side of the heart would come to be the only structure worth studying. The *vena cava*, in this view of the matter, is the seat of the primary morbid action. The heart is enfeebled by the malarious poison, in consequence of the deteriorated character of the blood, which has become black by the action of the miasm, and fails to supply the heart with its natural stimulus. Hence it accumulates in the large veins for the want of power in the heart to propel it forward. Congestion, in the view of those who adopt the hypothesis, is the great evil—the *causa sine qua non*—which being removed, the fever ceases.

To this theory, whatever may be the arguments in its favor, there is an objection that I cannot help regarding as fatal to it—which is, that the results of *post mortem* examinations do not reveal such a condition of the venous cavity. In a large majority of cases subjected to the test of the anatomist's knife, the liver has not been found in a state of congestion, or as exhibiting any morbid lesion. True, in nearly all fatal cases of fever, traces of congestion or of inflammation are to be seen in some of the abdominal viscera; and often, also, in the organs of the chest and of the head. But, so far as I have had opportunity to compare the histories of such cases, as given by the writers of all the conflicting schools, I have not been able to discover, that deep congestion of the vena cava, or of the portal circle, is by any means a uniform appearance. On this point, the testimony of Dr. Bailly is very full and striking.* This eminent physician visited Rome in 1820, 1821,

*Traitie Anatomico—Pathologique des Feivres Intermittentes, Simples et Pernicieuses. Paris, 1825.

and 1822, for the express purpose of studying the malignant fevers of that country. He has recorded the histories of thirty-six fatal cases of what he terms *malignant intermittents,* but which, in this country, would be called *congestive fever.* They exhibited, in their progress, every mark of deep congestion. Many of them terminated in twenty-four hours. The bodies of the patients were of an icy coldness. Some of the victims were comatose almost from the beginning, and others were foolish, like men intoxicated. In some, there was complete prostration of the muscular powers; but others were able to walk about their rooms only a few hours before death. In all, great coldness of the surface was present—a general shrinking of the features and extremities—the pulse was not to be felt at the wrist, and, now and then, not even in the carotids:—in all of which we trace the strongest resemblance to the character of our own congestive fever.

Of the thirty-six fatal cases recorded by Dr. Bailly,* the autopsy discovered arachnitis in 25; in 19, gastro-enteritis; in 18, splenitis; in 3, diffluent spleen, and in 3, ruptured spleen; in 13, cephalitis; in 7, gastritis; in 7, enteritis; in 5, *alterations of the liver,* of which, one was by inflammation, two by congestion, and two by putrilaginous softening. Pneumonitis, pericarditis, peritonitis, and inflammation in other parts were detected in many cases. From these statistics we see, that the tissue which suffered most frequently was the arachnoid membrane. If, however, we add the seven cases of gastritis to the nineteen of gastro-enteritis, we have twenty-six cases in which the stomach suffered. In every instance, inflammation in some part of the contents of the abdomen was discovered. In thirty out of the thirty-six cases, there was inflammation of the brain, or of its arachnoid membrane, conjoined with inflammation in some part of the digestive canal. The liver was found in abnormal condition in but five of the whole number, and congested in but two.

I will not undertake to affirm, that the experience of all

*Quoted by Dr. John Bell. Lectures. Phila. 1840.

writers on this subject is equally unfavorable to the congestive theory. Dr. Davis, for example, says, "dissection has shown that the organs primarily affected are the liver and the spleen. In subjects who have expired of this disease, even in the early stage, these viscera have always appeared to be materially altered in their structure."* Cleghorn says, "I have examined the bodies of nearly a hundred persons who perished in these fevers, and constantly found one or other of the adipose parts in the lower belly (the cacol, mesentery, colon, &c.) of a dark black complexion, or totally corrupted; the *vesica fellea* full and turgid, and the stomach and intestines overflowing with bilious matter; the spleen larger and sometimes weighing four or five pounds, and so excessively soft and rotten, that it had more the appearance of coagulated blood wrapt up in a membrane, than of any organical part."† Still, I cannot refrain from remarking, that the statistics furnished by Bailly appear to my mind to amount, as regards that theory, to an *experimentum crucis.* If congestion be the *foris et origo* of fever, how could it have been absent in so many of these cases? And if present, to hurry on the cases to the catastrophe, how are we to account for the disappearance of it, so soon after death? If the liver bear so much of the *onus* of febrile disorder, as is maintained by the advocates of the congestive doctrine, why do we find it, at the end of the worst cases, so often in a sound condition? Is not the inference irresistible, that, being sound, it is guiltless of the mischief, either as the primary cause, or as involved secondarily, and contributing to maintain the morbid action?

In Dr. Bailly's cases, the spleen, of all the organs, gave the most frequent proofs of congestion; for in twenty-three cases out of the thirty-six, it was in this state, or in a state of congestion conjoined with inflammation. The congestion, in a few instances, had progressed to the point of breaking up the tissues of that organ. Dr. Shapter, in the Library of Practical Medicine, also testifies to the frequency of lesions of

*On the Walcheren Fever.

†Diseases of Minorca.

the spleen in fatal cases of intermittent fever. According to all the authorities, indeed, this organ appears to be singularly often affected. But it were easy to show, I think, that this condition of the spleen is a consequence, and not the cause, of the febrile commotion. Who, for example, has not seen cases of enlarged spleen without fever? This viscus occasionally becomes so much increased in size, in consequence of repeated attacks of ague, as almost to fill up the abdominal cavity. And yet it is well known, that with all this congestion of the organ, fever is by no means the invariable attendant. That it sometimes is, will not be denied ; and that such a condition of the spleen adds to the difficulty of curing intermittents, all physicians have experienced. But it is as well known, that the chills may be checked, and temporarily, if not permanently, cured, while the spleen maintains its unnatural size. The two parts of the argument then, are, that the spleen may be enlarged without exciting fever; and that where they are conjoined, the fever may be relieved without removing the congestion of the spleen. And a distinct argument is, that the spleen is not invariably found in a diseased state at the close of congestive fevers. Why it should be often so is easily explained. During the chill, which precedes all fevers, the blood is accumulated in great quantities in the spleen by the very nature of its circulation. This organ is of a peculiarly loose, spongy, and vascular texture. In intermittent fever the febrile reaction is very great, and the engorgement of the spleen returning with every paroxysm, in the end, its structure can hardly fail to become altered. The morbid state thus induced in it, as an altered condition of any other organ, will react upon the irritated nervous system, and become, secondarily, the means of keeping up the morbid commotion. In like manner, congestion of the liver becomes a source of mischief, as must local lesions in whatever important organ existing.

According to this view of the subject, it is unphilosophical to regard hepatic congestion as the cause of fever, and, consequently, to address all our remedies to the liver.

I shall next inquire whether, by the same course of reasoning, gastro-enteritis, arachnitis, &c. are not excluded as the essential cause of fever. Some of these lesions attend all fatal cases of the disease, according to the experience of every pathologist, and probably contribute largely to the fatal termination. But do they not very often occur independently of fever, and run their course without developing that disease? Is it not a matter of daily observation, that gastritis, and inflammation of the intestines may exist, and yet every pathognomonic symptom of bilious fever be absent? In dysentery, cholera, gastritis, diarrhœa, &c. the symptoms are specific, but not those of miasmatic fevers. Arsenic or the other irritating poisons excite gastro-enteritis; but in such cases there are not the regular, well-marked paroxysms of fever. In dyspepsia the stomach is often in a state of sub-acute inflammation; yet this inflammation is far from developing an intermittent or remittent fever. Fever and such local lesions coexist; but these points of irritation, however they may aggravate and perpetuate the fever, cannot be regarded as the cause of it.

The following facts are equally unfavorable to the hypothesis under consideration: the irritation produced by introducing a catheter will sometimes excite a chill, followed by fever, which may be repeated for several days: I have seen a severe paroxysm of fever developed by keeping a tent in an irritable ulcer on the side for an hour, which could not have been distinguished from a regular intermittent. The shock of a cold bath has been followed by a chill and fever, which recurred at the same hour the next day. Bad cases of stricture and retention of urine are pretty uniformly attended by the phenomena of intermittents, so that we have a class of what are called urinary fevers. Richter cites a case in which the irritation of worms brought on the disease. The suppression of the catamenia and of other habitual discharges has been known to excite it. Shapter mentions the case of a girl nine years of age, in which a true tertain was clearly referrible to fright. In all these cases the fever is, indisputably, quite independent of gastric or intestinal irritation, and the facts show that an intermittent may arise and persist without such a cause.

We are assured, that "every system and every organ in the body may be, and frequently is, diseased during the course of fever;" and it is even probable that in a great majority of cases, "death is the result of one or many local inflammations."* But there is no constancy, no uniformity either in the seat or extent of the local disease. In one, we find the intestinal canal healthy; in another, the same parts present extensive disorganizations; and yet the symptoms in both, during life, were the same. Or we find, in other cases, the lungs inflamed, arachnitis, or rupture of the spleen; and in others, find all these organs in a healthy state. But if, as held by the advocates of the gastric theory, fever were a mere exponent of the inflammatory condition of the stomach and intestinal canal, we should as uniformly find such local lesions after the death of febrile patients, as we find disorganization of the lungs in death from pulmonitis; which, we have seen, is far from being the fact. Nor is this all, for we may have several patients presenting different symptoms, and yet on examination, *post mortem*, find the same morbid changes in all. In one, the phenomena of typhus may be present; in another, this condition may be slightly marked; in a third, it may be absent; and yet, in every one, we shall trace the same local lesion. Do we find, here, any of that constancy which subsists between cause and effect? We have similar symptoms excited by diverse lesions; and various phenomena excited by similar organic changes. To say that in such cases the fever is symptomatic of the local lesion, would be absurd. "A child," says Stokes, "is exposed to the contagion of small pox; for sometime nothing particular is observed; he then gets ill and feverish, and this is followed by an eruption of variolous pustules. Here we have a local disease consequent upon a circumstance affecting the whole system, and

*Stokes' Lectures, p. 409.

in this, as in local ulcerations attendant upon typhus, the local lesion is secondary. We might as well argue that the pustules were the cause of the symptoms in one case, as to say that the ulceration of the intestines was the cause of the other."*

Finally; the well known effect of bark in intermittent fever seems to me fatal to the doctrine of the physiological school. If we possess in any remedy a specific for any disorder, it is this article in recent cases of ague and fever. And yet bark is a tonic, a stimulant, and in genuine gastritis would be as mischievous, as in intermittent fever it is known to be salutary. How is it to be explained, that recent and acute gastritis is subdued by a remedy proved to be pernicious in other phlegmasiæ? I confess that the analogies cited by Broussais and his followers do not strike me as true. Chronic opthalmia, diarrhœa, and blennorrhagia, caused by stimulants, bear little resemblance to the inflammation that occurs during the progress of fever in the mucous membrane of the stomach.

The true account of the phenomena appears then, to be, that the repetition and excessive amount of the local congestions occurring in the cold stage of fevers, give rise to a hyperæmic condition of the affected organs. This condition, frequently excited, leads, at last, to local inflammations. New local inflammations are set up in various organs, and these, which, in the beginning, were only the effect of the disease, become, by reacting on the system, the cause of its continuance.† And hence, it appears, that in almost all cases of fever, there is a combination of the essential and the sympathetic fevers—the essential, the result of the first cause—the impress of malaria upon the nervous system—and the sympathetic, the result of the local lesions which arise during its course. So that, in the treatment of this disease, we are to bear in mind, not only the phenomena which seem to grow out of a general disturbance of the nervous system, but those

*Stokes. Op. cit. p. 414.
†Ib.

local inflammations also, which, springing up in its progress, serve to perpetuate the fever. We thus obviate the danger of death from the violence of the local affections, as also, the disturbance resulting from sympathetic irritation; whilst we reduce the disease to a state of the greatest simplicity, in whice quinine or bark may be given to arrest it. Upon the fact, that, these local lesions being removed, the accompanying fever often speedily subsides, Broussais chiefly founded his doctrine. But it has been shown that this was a hasty generalization. The true explanation of the fact is, that by subduing the local inflammation, we remove a focus of irritation, which opposed the salutary operations of nature.* After a few paroxysms of fever, we have ample reasons to suspect such local complications, and it is then found too late to rely upon quinine alone even in intermittents. Whether the inflammation be in the viscera of the abdomen, the thorax, or the cranium—and we have seen that all are occasionally involved—it constitutes a part of the morbid chain, and calls for a corresponding complexity of treatment. The antiphlogistic alone is not sufficient, as in pleuritis; nor that alone which acts upon the system.

The poison producing fever seems to exert its first influence upon the nervous system. The nerves of the cerebro-spinal axis are first disturbed. Hence the langour, head-ache, dulness, loss of apetite, disturbed sleep, and general *malaise*, the precursors of an attack of fever. The nervous system is marked by a peculiar law—that of periodicity, or intermission.† It is active, and reposes by turns. Repose is essential to its health. Intermission being an attribute of the tissue which sustains the first morbid impression, the same character is stamped upon the disease. All essential fevers exhibit this tendency, and in one type, the intermission is complete. All diseases located in the nervous system are intermittent, notwithstanding that the cause may be permanent.

---

*Stokes, Op. cit. p. 416.

†Dr. John Bell's Lectures, p. 550.

A spicula of bone, for example, irritating the brain will excite epilepsy recurring at distant intervals, though the irritation be always present. And, in like manner, coma, catalepsy and hysteria recur periodically, while the cause of irritation may be some permanent organic lesion. In fever, the system of nerves controlling animal life being depressed, all parts of the system—the skin, the viscera, and all the organs of circulation—are disturbed with it. For it is not mere depression of action; it is action also perverted. Congestions of the viscera, abdominal, thoracic, and cerebral, occur, which changes of temperature, irregularities of all sorts, fatigue, etc., especially favor; and these congestions being repeated, to be followed, still, by violent reaction, lesions at length are established in some system of organs, and become independent foci of irritation, reacting upon the cerebro-spinal axis, and increasing and perpetuating the morbid commotion.—That the intestinal canal should become most frequently the seat of these local inflammations would be inferred *a priori*, if post mortem examinations had not established the fact, from the circumstance of the extreme excitability of this tissue, and that it is peculiarly under the influence of aerial vicissitudes, of mental emotions, of fatigue, and of food and drink. All these, in fact, become the exciting causes of fever, when the nervous system is laboring under the effects of malaria. They often hasten the attack of which the premonitory symptoms had been for some days in existence; and by irritating the digestive apparatus they favour congestions and inflammation in that system.

Thus, to recapitulate, we have two classes of phenomena conjoined in fever: the first, irritation of the nervous system induced by the impress of malaria; the second, congestion or inflammation of some part, most commonly the mucous lining of the stomach and intestines, or of some of the associated organs, but often, also, of the lungs, and of the brain and its membranes. In the chill, the nervous system is depressed, and with it the action of the suffering organs falls below the healthy standard. Reaction follows the chill, and the diseased organs also reacting, irritate the nervous system still further, and excite the heart to more violent action.

### TREATMENT.

In the access of fever it is impossible to say what type it will assume. Remittent fever may be converted into an intermittent, by good management, in favorable cases, and intermittents often degenerate into the remittent form, or even assume the character of the formidable congestive type. In the same family, we meet with all the grades, from the mild intermittent to the congestive.

Dr. Cartwright assured the writer of this paper, that, in the yellow fever which prevailed in Mobile, New Orleans, and Natchez, in 1839, if, in the forming stage of the disease, viz; within an hour or two after the access of the chill, *ten grains of sulphate of quinine* were administered in a single dose, it often had the effect of arresting the fever or converting it into a mild and tractable form.* The same is true of the common intermittent; but, as in yellow fever, the remedy must be administered before the case is complicated by local inflammations. It was only while the fever was forming that the quinine possessed the power of subduing it; and it is only after the first few paroxysms, that this article can be relied on, singly, in chills and fever. After a few of

*Dr. Johnson in his work on Tropical Climates reports a similar practice as having been pursued by La Fucute, with singular success, in a malignant fever of Andalusia. "His plan," he remarks, "was to force the patient, if possible, to swallow six or eight ounces of bark within the first forty-eight hours of the disease. At the village of Los Barios, a few miles distant from St. Roque, ninety patients took the bark within the first eight hours of the fever, of whom *none* died, excepting one man carried off by a gouty affection. Of eight patients to whom it was administered between the eighth and tenth hour, all recovered. Of five who began between the twelfth and twenty-fourth hour, three recovered and two died. Of twenty who did not take it till the second day, thirteen recovered and seven died. Of seventeen who waited till the third or fourth day, eight recovered and nine died. And lastly, out of eighty persons who made no use of the bark, but took other remedies, only twenty-two recovered and fifty-eight died."

these commotions, it has been seen, the viscera become involved in disease demanding other treatment. In confirmed cases, purgatives must be superadded to the bark or quinine; and, not unfrequently, venesection, local and general, becomes necessary to subdue the local inflammations. Of the propriety of the last named remedy, the physician will judge from the violence of the paroxysm, the amount of local pain complained of, and the evidences, on examination, of inflammation in any organ. Dr. Stokes urges, with great emphasis, the importance of such an examination of all the regions of the body. "Here, gentlemen," he observes, "let me entreat of you to lay this down for yourselves as a rule never to be departed from, that before you prescribe the slightest medicine you first make an accurate and perfect survey of the state of the viscera. The whole nicety of treatment," he continues, "turns on this. If the case be one of the essential kind, we know the remedy which will answer, if not in all, at least in the majority of cases. If it be symptomatic, your treatment must be directed to the removal of the local lesions."* Quinine, aided, if complications exist, by moderate bleedings, and an occasional cathartic, of which calomel is the best, will not often fail to check intermittent fever. But the disease is known to be peculiarly apt to return again, on any slight exposure or indiscretion of the patient, and, after repeated recurrences, to induce an anœmic condition of the system. It becomes almost a law of the economy, and although often checked by quinine, as frequently appearing again under the most trifling irregularities. Such cases are familiar to all practitioners in miasmatic districts of country. Continuing, at intervals, through the winter, they break out in the spring, and persist during the summer. In such cases, bark and quinine cannot be depended upon; and, as in all similar states of the sanguineous system, in which the blood appears to be degenerated from a healthy condition and to be deficient in red globules, the preparations of iron exert the happiest influence.

*Op. cit.

Trousseau developes this principle fully in his recent treatise on Materia Medica. Dr. Stokes, also, gives his testimony to the value of iron, in such conditions of the system. I have relieved many of these obstinate cases of intermittent by the carbonate of iron, combined with a vegetable bitter. The ferro-cyanate of iron, however, is the form which seems to be best adapted to these cases. Stokes esteems the introduction of Prussian blue as a great improvement in medicine. He gives it in doses of from a "scruple to half a dram, three times a day."

The only other remedy of which I shall speak in simple intermittent is arsenic, which, according to M'Cullock, Sir Gilbert Blane, and others, may be used in some cases where bark has failed. This, Sir Gilbert found, was the fact in the fever which afflicted the British troops during the Walcheren expedition. In the fevers of young subjects who can with difficulty be brought under the influence of quinine or bark, I have often used Fowler's solution with satisfactory results. But where quinine can be given, and is borne by the patient, it is superior to all other remedies in recent cases; and has this advantage over arsenic, that it is uniformly harmless. Cases are on record, in which patients, who had used arsenic, fell into bad health, became weak, emaciated, and presented a remarkable derangement of the digestive organs, after recovery from the fever. Stokes relates instances of irremediable mischief from the long-continued use of this remedy. Such have never occurred in my practice; but the experience of so able a practitioner should induce caution in others. The dose of quinine commonly prescribed may be increased with advantage. Dr. Stokes prefers ten grains at a dose, once a day, to the same quantity in smaller doses during the day. I remember an interesting case, which goes to the same point. I had a patient, a young man from Alabama, who, having had a severe attack of pleuritis in the winter, fell into chills and fever in the spring. He tried the quinine in doses of one and two grains, and with them succeeded in checking the disease; but it still returned. In one of the paroxysms, perhaps the second of a third relapse, I gave him sixteen grains at a single dose, in the height of the chill. Warmth was soon restored; sweating came on without any well-marked febrile paroxysm; and the chills left him to return no more. I have alluded to a condition of the system, induced by repeated attacks of intermittent fever, in which the preparations of iron exert an excellent influence, but I should leave the subject very incomplete without a reference to those visceral obstructions that are so likely to arise in the progress of this disease, and which, in fact, constitute one of its chief sources of danger. Dropsy is a well known consequence of such obstructions, and it has lately become a matter of remark, that tubercular consumption is frequently developed in persons whose health has suffered from long continued intermittents. The danger of so formidable a disease should cause us to redouble our diligence to subdue the fever while yet in its recent and tractable stage. In chronic cases the patient should, if possible, seek a change of air, avoiding localities where malaria abounds; and, for the rest, should adopt all those measures which are calculated to restore the deranged viscera to healthy action, and impart tone and vigor to the system. For fulfilling the latter indication some practitioners prescribe sulphate of copper, in preference to the salts of iron, combining it either with quinine or some astringent bitter. In such cases, almost every thing depends upon the care and discretion of the patient, and without his faithful co-operation relapses will continue to take place, and, at length, some of the dreaded sequelæ will make their appearance, in spite of all the resources of the physician.

Intermittent fever, now and then, assumes a form in southern climates, in which other modifications of treatment become necessary. I allude to what is called in this country, congestive fever, but which seems to have been long ago described by writers under the name of "malignant intermittent." Alibert gives an accurate description of congestive fever under the title of "algid" intermittent. From the descriptions given by Senac, Lancisci, Lind, Pringle and Cleghorn as well as by Bailly more recently, of these intermittents, it is clear to my mind, that they are identical with the formidable fever of our region. A more graphic account of our congestive fevers could not be found than that contributed by surgeon Shields in Johnson's work on Tropical Climates. "The patient," he says, "on the first attack frequently falls down and is insensible during the paroxysm, his body covered with cold clammy sweats, except at the pit of the stomach, which always feels hot to the palm of the hand; the pulse is small and quick. The length of the paroxysm varies from six to eighteen hours, and was generally succeeded by cold rigors, very often low delirium, preparatory to the next stage or paroxysm of the fever. The intellectual functions now become impaired, the patient not being at all sensible of his situation or of any particular ailment. If the patient be asked how he is, he commonly answers 'very well,' and seems surprised at the question. This is a very dangerous symptom, few recovering in whom it appeared. A great proportion changed in a few days to a bright yellow, some to a leaden color; other cases terminated fatally, in a very rapid manner, too, without the slightest alteration in that respect. Generally, however, the change of color indicated great danger. In some a purging of vitiated bile occurred; in a great many a torpor prevailed throughout the intestinal canal; rarely did any natural fæces appear spontaneously."

Lind describes the following case as a malignant intermittent, which answers fully to the symptoms of congestive fever:

"A young gentleman was seized with a fit of an ague, and in half an hour became delirious, then comatose, and at length speechless. Finding him in this state, I ordered a blister to be applied to his back, and a cordial julep with salt of hartshorn to be poured by degrees into his mouth. In two hours afterwards, upon recovering his senses so as to swallow with ease, I ordered him two ounces of tinture sacra, and then as soon as the fever and sweat had abated, without waiting for the complete effect of the purge, half a dram of bark every four hours. He began the use of the bark three hours after he had taken the tinct. sacra; but before he had taken five drachms of it, he was seized with a second fit, and in like manner became delirious, comatose, and speechless. Sinapisms were applied to his feet, and other irritating applications used, until the fever was terminated by a plentiful sweat.—Thus, having twice narrowly escaped dying in the fit, a dram of the bark was ordered to be taken punctually every hour. He soon took two ounces of it, which had so happy an effect, that the fever left him entirely, and he was quickly restored to perfect health."*

I will add one case more of algid fever, as given by Dr. Bailly. The subject of it was a man æt. 60 years, of thin habit of body, but healthy up to the time of the attack, 18th August.

"He had a paroxysm of the common kind, which went off in a sweat. On the 19th he was brought to the hospital, and had a return of the fit, in which he complained much of inward heat. Expression anxious; the features were in a maneer flattened on the bones of the face; the complexion was natural; look heavy. Evening: decline of the fit, skin moistened with a viscous and cold sweat; pulse small and frequent; agitation general; pain at the epigastrium; tongue red, but moist—no thirst. (Half an ounce of bark.) Throughout the night the skin was moist. Patient vomited the bark.

"On the 20th of August, in the morning, there was a remission of fever, the pain had disappeared, and the countenance was tranquil. About noon, a fresh paroxysm came on, and although a great heat succeeded the chill, the extremities remained cold, and the skin was covered with livid spots. On the 21st, general calm, but the extremities still cold; pulse small and frequent. Towards noon, there was a return of fever, preceded by chill; exacerbation of the preceding symptoms. The patient does not feel the coldness; he is in a measure benumbed and torpid. (Bark: an ounce to be taken through the night.)

22d. Skin less cold; pulse small and frequent; a viscous sweat over the whole body; look heavy. (Two ounces of bark.) At 10 o'clock, return of the paroxysm; pulse not to be felt at the arm; beats 140 at the crural artery. An icy coldness of the extremities; abdomen flattened, even con-

*Essay on diseases incidental to Europeans, in Hot Climates, &c.

cave, its parieties applied as it were to the spine; pain of the stomach, agitation, anguish: the patient who has never lost his understanding, is in a state of torpor, which barely allows of his answering any question. The complexion is natural. (Twelve leeches applied to the epigastrium; blisters to the arms; bark, three ouuces in powder to be taken during the night. He has vomited the bark.)

"23d, A well marked remission. Towards nine o'clock, return of the cold, which is like that of marble; pulse imperceptible, artery beats 144 at the thigh. Pain of the stomach more intense; anxiety, great torment, eyes sunken. The cold, which hitherto had been confined to the extremities, extended to the shoulder and the pelvis. The head was cool, the thorax and abdomen not of their natural temperature; thighs icy like the legs. Evening; same state; he did not feel the cold of his legs, but was aware when touched by another that the skin of the latter was warmer than his. Pain of the stomach incessant. Lies on the back. Death at 3, A. M."

I will now briefly compare the history of congestive fever with the description of these cases. Dr. Russel thus writes of this fever:*

"There is nothing in the first chill or fever calculated to alarm; but the second or third may indicate the greatest danger. The chill is not generally very distressing, but the excitement upon reaction is excessive—the pulse is full, frequent, and resisting; the skin hot; pain in the head intense; the eyes often red; thirst for cold drinks urgent; violent throbbing of the carotids—in fine every symptom of a most malignant fever is present. Upon the decline of fever, a profuse, cold, clamy sweat breaks out, but affords no relief. The pulse rapidly sinks, becoming sometimes imperceptible. The whole surface is cold and shrunken, but especially the face, giving to the countenance a ghastly expression. Great distress, referred to the chest and epigastrium; oftentimes a burning heat, and superadded is a restlessness, which the patient cannot control. Respiration hurried and difficult. In some cases constant vomiting, or repeated ineffectual efforts; in others, hypercatharsis, which usually ensues after the exhibition of a hydragogue. The strength of the patient is of-

*Transylvania Journal, vol. 6, p. 90.

ten astonishing—he is able to rise at pleasure from his bed—to change his position from place to place; in fine, to control every muscle of his frame."

The following is a case of congestive fever, reported by W. J. Johnson in the first volume of the Southern Med. and Surg. Journal: About sunrise on a morning in August, 1835, a negro man, aged 30, was attacked with pain in the head and shivering. He was ordered to bed, and wraped up warm, and a bowl of hot sudorific tea was given him. Patient complained no more, and lay in bed perfectly calm and quiet; breathing laborious; eyelids open, and eyes fixed; made no reply when spoken to; extremities cold and clammy; pulse slow and struggling. Death in a few hours.

The same writer details the particulars of another case:

"A boy at school, æt. 14, in the midst of apparent good health, was seized with congestive fever in such a degree that he was thought to be dying. He was bled, and vomited—half-digested chesnuts were thrown up. Cups were used, with sinapisms to his extremities, and a blister to the neck. He continued in a state of collapse. By the use of calomel, enemas, cupping, and stimulants, a large quantity of dark, vitiated, offensive bile was brought away from the bowels. After this, he became sensible and expressed himself better. Reaction was considerable, and the antiphlogistic plan was pursued for several days. His friends entertained the strongest hopes of his recovery, when on a sudden he complained of acute pain in the head—went delirious, and seemed for some time to be laboring under an attack of phrenitis. These symptoms were attended with convulsions and other nervous symptoms. Blisters were re-applied to the head and behind the ear; but notwithstanding this, he went in a fatal collapse, from which he never recovered. The pupils of his eyes were widely dilated, insensible to light; picking at the bed clothes; low, muttering delirium; incoherent speech; *subsultus tendinum;* involuntary evacuations, were the symptoms which closed the scene."

I will not multiply instances; but I cannot forbear expressing my belief, that any physician who will carefully compare what he has seen of congestive fever with the histories given by European, and especially Italian writers, of the malignant intermittents of miasmatic regions, will come to the conclusion, that the two diseases are identical. In the congestive fever of the South, therefore, I conclude that we have the algid fever of Alibut—the pernicious or malignant intermittent of Senac, Lancisi, Lind, and other old writers on the diseases of the tropical climates. It is proper, however, to remark, that remittent as well as intermittent fever is subject to degenerate into this malignant type, and in treating of the cure I have not pretended to distinguish between them, regarding them all as modifications of bilious fever. Congestive fever, I have said, calls for a modification of the treatment usually successful in the simple remittent or intermittent. That modification has been introduced by the practitioners of the Valley of the Mississippi, and if judiciously adopted and pursued, promises to rob this fever of a large share of its terrors. It consists in the *cold water dash*, as in cases of asphyxia, and in liberal doses of sulphate of quinine. The latter remedy given in the forming stage of the disease will often convert it into a simple intermittent. It is to be given in ten grain doses, and repeated according to circumstances. Of the application of cold water in asphyxia of such fevers, with signal success, we are in possession of much testimony from practitioners in various parts of the South-west. Among the first to adopt the practice, I believe, was Drs. Fearne and Erskine, of Huntsville, Alabama; but without being acquainted with the results of their experiments, Dr. Addams, of Cynthiana, Kentucky, resorted to it, with the happiest results, in the asphyxiated stage of cholera. Dr. Russel, in the article referred to, has recorded some highly interesting and instructive cases of recovery from congestive fever under the use of the cold dash. I cannot better illustrate the practice than by transcribing some of these cases.

His second case was that of a man whom he found in the greatest state of restlessness, complaining of a sense of excessive internal heat.

"His pulse was barely perceptible, skin cool and wet with sweat, respiration impeded and frequent; there had been no evacuation from his bowels, notwithstanding he had taken, according to the estimation of his friends, sixty grains of calomel. This being the third case I had seen, and my confidence in the efficacy of cold water being unbounded, I without delay made the application. As usual, the relief was instantaneous, and so agreeable was it to the patient, that for the remainder of the time he was confined, he kept by his bed-side a vessel of water which he used at his pleasure according to the suggestion of his feelings. Reaction was thus completely established, and with mild cathartics and quinine he was entirely restored to health."

Dr. Erskine details the particulars of a case, the subject of which was found in the third paroxysm of regular intermittent fever of the tertian form, which had been neglected. The cold, clammy surface, feeble pulse, great restlessness and precordial distress were present, as in all such cases. Cold well-water, rendered colder by the addition of table-salt, was dashed on the naked surface. He feel asleep soon after being put to bed, and had ten or twelve hours rest. Reaction was complete next morning, pulse slower and fuller; no return of the clammy sweat. "He recovered in a few days upon the use of bark, with occasional doses of mild aperient medicine."

A second case reported by Dr. Erskine is one of algid intermittent. The subject, a female, had the symptoms of simple intermittent; but in the second paroxysm, symptoms of a malignant character occurred. A cold, clammy sweat appeared on the extremities, accompanied with great restlessness and anxiety, for which Dr. E. at first tried sponging with cold water, as the friends objected to the cold dash; but the patient growing much worse in twelve hours, he had recourse to the affusion. When he resorted to it, the extremities were cold and shrivelled, pulse feeble, features contracted; great restlessness was present, with considerable alienation of mind. The water was poured on her body from a pitcher, and being wiped dry she was put to bed, where she slept for an hour or two. The cold sweat was then found to be returning, and the cold water was again applied. The renewal was found necessary three or four times. A few doses of calomel and quinine completed the cure.

The last case reported by Dr. Russell is in the language of Dr. Fearn, and is highly instructive:

"Sylvia, a favorite mulatto servant, belonging to Dr. David Moore, was attacked with intermittent fever on the 11th of September. I saw her on the morning of the 16th, and found her in the following condition: Her whole body shrunk, cold, and bathed in a clammy perspiration—skin shrivelled, as if she had been for some time immersed in water. The extremities were of a marble coldness—some restlessness, and occasional sighing, but without any distinct pain; mental faculties unimpaired; pulse scarcely perceptible at the wrist, and beating 130 in a minute; tongue moist; cold drinks alone were craved; every thing warm or stimulant was loathed. I was informed that the first paroxysm had gone off in the usual manner, with perspiration, and was followed by a complete intermission. On the 13th, a second paroxysm came on; the extremities continued cold for some time after the body was hot, and the fever did not give place to an entire intermission, as it had done two days previously. On the 15th some fever continuing, an emetic of ant. tart. was administered. It operated well, but not inordinately, both as an emetic and cathartic. The fever subsided rapidly, perspiration came on, and continued through the night, increasing in quantity, and assuming a more cold and clammy character, until she was reduced to the situation in which I found her next morning."

Cold affusions were ordered, to be repeated every two hours, but as the prescription was left to be executed by the overseer, the physician found, in the evening, that the bath had been given but twice, and that the situation of his patient was in no way improved. On his visit in the evening the affusion was again made, and by next morning the danger had passen. She had slept well—warmth was restored. Quinine and bark, with mild laxatives, were the only remedies subsequently used. The paroxysm did not return, and the convalescence was rapid.*

*Trans. Jour. Med. loc. cit.

To the efficacy of the cold affusion in the collapsed state of congestive fever, I will only add the testimony of Dr. Keller, of Alabama. He says:

"I have seen it tried often, and in two-thirds of the cases it proved entirely successful. One case I recollect—that of a favorite servant; the family had despaired of her life, and had removed her to an adjoining room to die. She was entirely pulseless, and had been so for hours; the cold dash was applied twice in an hour, and in three hours the excitement was so great as to require the free use of the lancet—in a few days she was as well as ever." He adds: "If cold water fails to excite in this stage, you may consider the case of your patient hopeless."

Dr. K. has found it far more effectual in rousing the system from the state of asphyxia, or collapse, than the most powerful stimulants.

It is curious to remark, how exactly this is in analogy with the action of the same remedy in asphyxia from other causes. In poisoning with the mephitic gases, and in cases of drowning, or of syncope from the effects of lightning, cold water in the form of affusions is well known to be the most efficient remedy. The same is also true of poisoning by opium; and late experiments go to prove that in the asphyxia induced by an over dose of hydrocyanic acid it is the most certain restorative. So that, on this point, we seem to have arrived at a general truth—to have established a great and valuable principle; and if future researches should confirm it, cold water will become the remedy in all aphyxiated conditions of the system, whether induced by lightning, the noxious gases, an over dose of arsenic or prussic acid, or occurring in fever or cholera. The experience of Dr. Addams in the collapse of cholera has already been alluded to, and his paper on the subject contains the history of a number of cases of the highest interest, to which, as being analagous to cases of congestive fever in the same stage, I will refer more in detail.

His first case is that of a man aged 30, of robust constitution. He had been affected with choleric symptoms for four or five days. Collapse supervened on the fifth. His skin was icy cold and bathed in perspiration—pulse nearly imperceptible, breathing oppressed, tongue cold, spasms, eyes sunk, "countenance collapsed." Vomiting, and watery discharges from his bowels copious and frequent. Recovered under the cold water affusions, with a few doses of calomel and quinine.

Case 2d.—A little boy, aged 11, had all the above described symptoms. Other remedies failing to excite, the cold affusion was applied, once and again; reaction occurred, and he got well.

In the third case the termination was fatal, the patient, a woman, aborting, and sinking twenty days after her attack. The cold water recovered her from the state of collapse, and her death was clearly the consequence of the abortion. The child had been dead for several weeks, from appearances indicated.

The next case was one of deep collapse. It terminated favorably. The cold water was repeatedly applied, always restoring warmth and exciting the pulse.

Dr. A. refers to many cases treated successfully by other physicians in the same way. After free depletion by venesection, purgatives and emetics, he deems the remedy inapplicable.*

It would be difficult, I think, to find in the annals of Medicine, more unequivocal testimony to the efficacy of any remedial agent, than has here been brought forward in favor of affusions of cold water in the asphyxia of congestive fever. I shall add nothing to these details which already, perhaps, have been sufficiently extended.

Before taking leave of the treatment of congestive fever, I will allude briefly to the views of a late writer on the subject. Dr. Hardin, of Kentucky, reported some cases in the first volume of the Louisville Journal of Medicine and Surgery, marked by certain peculiarities, which terminated fatally. In a late number of the Western Journal of Medicine and Surgery he has extended his remarks, and has added two fatal

*Trans. Jour. Med. vol. 8, p. 61.

cases which exhibited the same peculiarity. This peculiarity consisted in copious evacuations from the bowels of "mucus, as green as wheat, inodorous, exceedingly acid, and incapable of communicating any tinge to water." He treated these cases in the usual way, with calomel, v. s. blisters, ipecacuanha, pulv. antimon. &c. Gangrene of the mouth and gums came on, with fatal symptoms of collapse. He regards the *green, mucus discharges* as pathognomonic, and insists, that whenever they appear, calomel is poisonous, inducing with fatal certainty gangrene of the mouth. In such cases he applies sinapisms to the whole extent of the spine, upon the principle of arousing it to action, and thereby invoking secretion from the abdominal viscera. And this practice appears to have succeeded admirably in his hands. He relates the histories of several remarkable cases, and states that he has had much experience in the application of the remedy. The following is the sixth case in Dr. Hardin's paper:

"Sept. 1st. 1838, I was requested to visit a youth aged 12 or 13 years, of good constitution, who had been sick some twelve days. Symptoms—expressed himself as being free from pain; tongue dark red and very dry; skin rather hot; pulse too frequent and sufficiently full; restlessness very considerable. He had been in a similar condition from the commencement of the attack; had taken 25 grs. calomel every night, with rhubarb on the following morning when the calomel failed to operate. Discharges represented as having been consistent and bilious from the beginning. The same treatment was continued for one night longer.

"Sept. 3d. Gangrene of the gums apparent; surface cool; pulse small, weak and frequent; alvine evacuations represented as being still consistent and bilious; but upon inspection were found to be a dark green mucus. Spinal irritation with mustard seed was used every twelve hours, and the bowels moved with rhubarb. Under this treatment the patient recovered, but was considerably endangered for some days by gangrene and hemorrhage from the gums."*

In all cases of this fever (congestive) of ordinary violence,

*See No. xiv. of this Journal.

Dr. H. found the respiration laborious, with feeble radial pulsation, cool surface and extremities, and an intolerable sense of weight at the epigastrium. He had in vain attempted to rouse the circulation by synapisms and warmth to the extremities; but, applied to the spine, they have produced marked advantage. The extremities grew warm, the præcordial distress is removed; in other words, the balance of the circulation is restored. Besides the irritation over the spinal cord, Dr. H. gave rhubarb as a purgative; but no calomel. He relies upon the sinapisms, aided by ten grains of Dover's powder and five grains of camphor, to prevent the paroxysm.

I have gone somewhat fully into an explication of Dr. Hardin's practice, because it is novel, and involves a principle which, if confirmed, will prove a highly important one. That there are cases in which calomel is unequivocally pernicious, may well make those pause who use this remedy, indiscriminately and perseveringly, from the beginning to the close of bilious fever in all its grades. If Dr. H. has hit upon the distinctive feature of such cases, he has discovered a most valuable fact in practical medicine. I can only add that he is a gentleman of excellent moral character, whose accuracy and faithfulness in the statement of facts may be fully relied on. And in the present case he reports, that his experience has extended through two years, and that he has tested the practice in a multitude of cases.

In support of the liberal use of quinine which I have proposed, much testimony may be adduced. I have referred to the remark of Dr. Cartwright concerning its utility in the access of yellow fever. Dr. Perrine, twelve years ago administered the medicine in such doses as to amount to a dram in a single intermittent.* Dr. Monette, both in the American Journal of the Medical Sciences, and in the Western Journal of Medicine and Surgery has given his favorable opinion of the practice. Dr. Drake says he has been in the habit of giving it in doses of ten or fifteen grains.† Dr. May reports some

---

*Western Journal of Medicine, vol. xi.
†Ib.

interesting cases in which it acted benignly in such doses. In his own person he used fifteen grains of quinine, during the fever, which left him while taking it. "I passed the day," he says, "without the recurrence of fever; was affected with some degree of stupor, ringing in my ears and deafness; but with no other uncomfortable sensation. A dose of calomel taken about 8 o'clock, A. M., brought off evacuations of the consistence of black clotted blood."* He recovered, using quinine in smaller doses, with calomel and rhubarb. Dr. May found that given in the febrile paroxysm, it uniformly reduced the pulse. Such is the experience of Dr. Thomas Fearn, also, who gives a striking case in the 9th volume of the Transylvania Journal of Medicine. The patient labored under bilious fever, having had repeated attacks of intermittent fever in former years. It was in the advanced stage of the disease, and Dr. F. believed his patient would die in the next paroxysm. He gave quinine in doses of thirty-two grains at the occurrence of the apyrexia, but while the pulse was still a hundred in the minute. In an hour there was a diminution in the frequency of the pulse—"the invariable effect," he remarks, "of a large dose of quinine." Another dose of the same size administered in an hour was attended with a still further reduction of the pulse, and a "ringing in the ears." A third dose was given, making ninety-six grains in three doses. The patient recovered, Dr. Fearn's ordinary dose of quinine is twenty grains.†

From all this—and it were easy to add much to the same effect, if time and space permitted—it is clear, that quinine is to be regarded rather as a sedative than a stimulant, conforming, thus, in character to opium, which stimulates in small doses, but in large doses calms and subdues nervous and arterial excitement. Quinine, consequently, may be administered as well in the remissions, as in the intermissions of fever. This is the conclusion, at least, to which the writer of this essay has come, and the practice he would earnestly recom-

*Transylvania Journal, vol. x

†Transylvania Journal, vol. x.

mend to the consideration of the profession. This preparation of bark appears to be a *febrifuge*, in the true acceptation of the term—preventing the chill, and moderating and abridging the paroxysm of fever. I would give it at the access of fever, while the disease was yet in its forming stage, and I would watch the earliest remission, and administer a dose of ten or fifteen grains, with a view of converting the subsidence into a complete intermission. And from all the experience I have had, as well as from what others have written concerning this practice, I am convinced that it is the best mode of treating fever. In saying this, however, I am far from meaning to convey the impression, that, in the treatment of fever, I should rely exclusively upon quinine, or upon it in conjunction with all the remedies heretofore enumerated. I mean simply to say, that I am constrained to regard it as a most important improvement upon the system too generally confided in—that of purgatives and the lancet.

Of the lancet I have already spoken. It should be used wherever we have reason to suspect local lesions, and it should be employed, also, in cases of excessive arterial action to obviate the danger of such lesions. But I should deem it a useless waste of time to dwell upon the value of a remedy, the indications for which are so familiar to the profession. That it has been abused, and that it may be carried to excess, no one can question, any more than that it is imperiously called for in many cases of fever. Marshall Hall has well described a morbid state to which excessive venesection gives rise. We see it in the nervous irritation, the throbbing temples, headache, &c. with which females suffer who have been the subjects of profuse uterine hæmorrhage. But in describing the circumstances which should regulate the employment of the lancet, I should be but repeating what is fully and clearly laid down in every standard work which treats of fever. Nor shall I dwell at greater length upon the value of local bloodletting, which, by the universal consent of the profession, is placed at the head of the means for subduing local inflammations. Leeches are more eligible under certain circumstances, but cupping is to be preferred in a majority of cases, as being more under our command, and enabling us to adapt the bleeding to the violence of the affection. I esteem the general use of this measure by the profession one of the greatest improvements in the practice of this country. As a mean of controlling local disease, it exceeds in power all other remedies, at the same time that its action is immediate and clear, and its application attended with but little pain. It is never to be omitted where the disease shows a disposition to fix upon particular organs.

It is difficult to speak of purgatives in fever without entering into discussions and details which would extend this essay beyond all reasonable limits. I shall, therefore, confine my remarks to a few points upon which my experience enables me to speak with confidence. I will not institute a comparison between the value of cholagogue and hydragogue cathartics, nor dwell upon the fact, so universally admitted, that the procurement of dark, consistent evacuations is a most favorable circumstance in cases of bilious fever. Neither shall I stop to inquire in what way the purgatives, usually styled bilious, exercise their curative influence—whether by promoting the secretion of the liver, or by removing the local lesions of the alimentary canal which are known to maintain febrile excitement. The statement of a few facts, however, bearing on this point, may be proper. First, then, it would seem to be unphilosophical to address our remedies exclusively to an organ which post mortem examinations have proved to be frequently free from disease; and equally so to infer, that, because purgatives act beneficially, they do so by disgorging the liver of its vitiated bile. That calomel, which exerts a decided influence on the liver, is a most valuable remedy in fever, I freely admit; nay, I am ready to maintain, that it is indispensable in the management of the fevers of the Misssisippi Valley. But it does not necessarily follow, that it relieves the patient by its action on the liver, which may be in a healthy condition.

The second fact upon which I would lay a good deal of stress is, that calomel, which may stand for the class of purgatives, has been proved by many experiments to be quite the opposite of a stimulating, or irritating medicine, when applied to inflamed surfaces. In the person of the patient of Dr. Beaumont, this fact was clearly established. When the stomach of Saint Martin was seen to be red and dry, and he had no appetite, a few grains of calomel introduced through the orifice in the abdomen had the effect, in a short time, of removing the hyperœmia, and allaying all the symptoms of gastric disturbance. The same remedy we apply to external surfaces in an inflamed condition. We give it in dysentery with advantage, where the large intestines are known to labor under inflammation. It is a remedy of most unequivocal value in croup, the essential feature of which is high inflammation. We give it in cases of excessive gastric irritability, where no other medicine can be retained, and we find it to check and relieve the vomiting and nausea. But why multiply examples? All the facts connected with the operation of calomel prove it to be a sedative, allaying gastric irritation, favoring sleep in the sick and watchful, curing a bad cough, and relieving the heat and pain of gonorrhea. That such is its character no physician can doubt who has watched its action upon young children, and seen it induce sleep like an opiate. And as a sedative no doubt it is, in part, that it brings relief to the febrile patient. Acting directly upon the inflamed surfaces, it removes one of the conditions by which the febrile action is perpetuated. But the liver and other abdominal viscera being often though not uniformly in a state of congestion, it relieves this state of things by promoting their secretions. Calomel, therefore, is well retained as one of the most efficient remedies in fever, although we reject the theory of hepatic congestion upon which it has been used to so great excess.

In point of activity there is no great difference between a large and a small dose of calomel. The article is nearly insoluble, one part requiring two thousand parts of water to dissolve it, and, consequently, when administered in large quantities, much of it passes unchanged, but partially altered in chemical composition, as I have observed. I have known a patient to take sixty grains of calomel, and have but a single moderate passage from it; and a week afterwards have known the same individual to be purged six times by ten grains of this medicine. Of course, his system was in a different condition at the two periods; although the difference was not manifested by the symptoms, for he was laboring under diarrhœa each time. But all physicians must have remarked, that the effects of this medicine are by no means proportioned to the dose of it, and that one or two grains of it will often excite the bowels quite as freely as a scruple. Some writers maintain, that it is more likely to irritate the stomach and bowels in small than in large doses.

If these views be correct, very large doses of calomel, to say the least, are unnecessary. It is also unphilosophical, upon these principles, to increase the dose with the evidences of intestinal torpor, and to depend upon curing the disease by purging from the liver. If a dose of ten or twenty grains fail to operate, it is a proof that the remedy is not in harmony with the prevailing state of the system. The excitement may require to be moderated; and, then, v. s., cold water, tart. emet. or ipecac., in small doses, will cause the purgative to act well. Often its action will be promoted by combining it with quinine. In favor of this practice I might cite much authority. It may be found in all the American journals of medicine. By the use of the latter article in large doses, the system may be brought into a condition in which a few grains of calomel will produce satisfactory purging. And finally, according to the experience of Dr. Hardin, in one condition of the spinal cord, the stimulation of sinapisms, applied along its course, will promote the desired operation. Far, therefore, from relying upon calomel alone, I cannot too strongly insist upon aiding its salutary action by the lancet, when indicated —by cupping, and leeches—by cold water, copiously used, externally and internally—by quinine, given so as to anticipate the paroxysm, and to shorten it—or by the application of excitants, in the apyrexia, or remission, to the spinal column, as the state of the patient may point to the propriety of one or the other of these measures. I am sure we have relied quite too exclusively upon this potent remedy in the treatment of our fevers.

A few years ago it was the practice to hasten the operation of calomel, when slow, by the administration of the neutral salts or castor oil. Dr. Rush, it is well known, used calomel and jalap combined, in doses of ten grains each. But since the appearance of epidemic cholera in this country, and probably even a year or two earlier, a disposition to serous evacuations has been remarked, in our fevers, which such purgatives promote. Hence, for a number of years, they have been pretty universally discarded. But this feature in the fevers of the South is gradually disappearing, and the saline purgatives may be restored to favor. As less likely to excite watery purging, aloes, rhubarb, extract of the juglans cinerea, and scammony, are both adapted to cases in which there is a proneness to such evacuations. These are to be administered in from eight to twelve hours after the calomel, should this fail to act in that time, and must be repeated, if the first dose is not sufficient. But here, again, I must bear my testimony against that practice, which consists in repeating dose after dose of such articles, without any attempt at bringing the system into a state favorable to purgation. Incredible doses of jalap, rhubarb, scammony and aloes are sometimes given in fever without purging the patient. In such cases the liberal use of cold water will frequently relax the bowels, especially if it be aided by the abstraction of a little blood, or by minute doses of tartar emetic. And where the stomach is so irritable as to reject all medicines, nothing will be found so soon to quiet it as the cold bath, and the liberal use of ice. I prefer giving a dose of calomel at bed-time, and securing its action by suitable adjuvants next day; and then waiting till evening before repeating it. Some hours are necessary to enable it to exert its whole beneficial influence upon the system; and once in the four-and-twenty hours is as often as it has been my practice to exhibit it. At the same time, I will not undertake to say, that there may not be cases in which it is proper to administer it more frequently. But I greatly fear, that by those who hold, that the only way of curing fever is to purge from the liver, it has often been pushed too far; an error which has contributed to excite great prejudices against it in the public mind, and to give a class of empirics a temporary advantage over the regular practitioner. The danger of ptyalism—very great in children—is not to be forgotten where it is necessary to give calomel day after day for a long time. Nor should we lose sight of the fact, that there are morbid states which particularly favor this mischievous action of the medicine, and that many constitutions are peculiarly susceptible to it.

If, therefore, there be danger in large doses—danger of salivation, and of fatal gangrene of the mouth—and if small doses are as active, but not so likely to salivate, as large ones, why should we persist in the employment of the latter? If we can avoid the danger, and at the same time accomplish our object, by combining the calomel, or by varying our remedies to suit the varying indications of the disease, nothing is plainer, than that the moderate use of this formidable remedy is alike a matter of duty and good policy.

Of the subordinate remedies called for in the cure of fevers, I shall not speak at great length. I do not estimate the value of emetics highly, nor consider them as admissible in cases of gastric irritability, which symptom I have seen them seriously aggravate. Upon the whole, unless there are irritating ingesta to be removed, I am not sure that we should lose much by rejecting them in the treatment of our fevers. Yet, while I express this as the result of my own experience, I must admit that high authority can be found for the use of them. I well remember the success of the late Dr. Wilson Yandell, one of the earliest practitioners of Tennessee, whose uniform practice it was to administer an emetic, in the first stage of the attack. He held, as Rush also maintained, that by this course he frequently cut short the fever, or, at least, rendered it more mild and manageable. Graves says, tartar emetic frequently succeeds in cutting short, or removing febrile symptoms, and it is evidently one of his favorite remedies in those cases of typhus, where there is undoubted evidence of determination of blood to the head, producing headache, loss of sleep, and delirium. But this admirable author truly remarks, what must have struck all physicians of observation, that every epidemic is peculiar and distinct in its nature, and that each, consequently, requires a peculiar mode of treatment. The fevers which have prevailed in the Valley of the Mississippi for the last fourteen or fifteen years have been marked by a tendency to congestion, in some seasons, and, in others, by an irritability of the stomach, forbidding the use of emetics. This, I am persuaded, is the general experience of the physicians of this region of country. I have long been convinced, that emetics exerted a most baneful influence in the case of the excellent physician above alluded to, whose premature death was occasioned by an attack of autumnal fever, in which he took tartar emetic more than once. After the operation of the last dose, his disease assumed the formidable congestive type. But I entertain a very different opinion of the virtues of cold water, as a drink and external refrigerant, in the hot stage of the disease. Indeed, I could hardly speak too warmly in praise of it, whether the comfort it gives to the patient be regarded, or the effect it exercises in moderating the violence of febrile action. Yet I do not think it admissible to dwell upon a remedy the virtues of which all practitioners are beginning to admit, and which must become the most popular of all our remedies for fever. Cold water is hardlv to be ranked among the subordinate means for controlling febrile action. It deserves, rather, to be classed among the most efficient of the measures. Fortunately, it is one most consonant to the feelings of the patient. The history of practical medicine affords not a few instances in which patients have recovered, by violating the precepts of misguided practitioners, and indulging freely in the use of cold water. Ice is even more grateful than cold water from the spring, and in cases of great gastric irritability is one of our best remedies. Great is the relief I have often witnessed, in such cases, from allowing the patient to swallow little pieces of it every few minutes. In cholera infantum and dysentery, as well as fever, I have repeatedly seen it allay nausea and vomiting in the promptest manner.

While on the subject of cold water I will mention, that I have experienced admirable effects from the cold dash in cases of fever marked by obstinate determinations to the brain. The patient is to be placed in the hot bath, and while in this situation cold water is to be poured on his head from the mouth of a pitcher, or the spout of a coffee-pot.*

Opium may be given in the cold stage of intermittent fever without aggravating the fever, and with the good effect of abridging the cold paroxysm.

Blisters are indicated in fevers under a variety of circumstances, but it is particularly as stimulants, in the latter stage, or as revulsives, where local determinations occur, that they are to be used; and in such conditions their effect is often most salutary. Who has not seen them relieve the pain in the head, chest, or abdomen, sometimes persisting after free depletion, like a charm? No doubt they are often applied too early, in which case they increase the febrile excitement. They are not to be resorted to until after the violence of the disease has been subdued by evacuants. If, afterwards, local affections are manifest they are to be employed, unless cupping should be more clearly indicated.

The pulvis ipecac. compos., as a diaphoretic, may be employed in the advanced stage of fever; and the pulvis antimonialis is admissible at an earlier period. Tartar emetic in minute doses, with a view to diaphoresis, is a favorite prescription with many able practitioners.

But having pointed out the cardinal remedies in the cure of fever, I shall not attempt to enumerate the details of treatment; but shall only add, in conclusion, that much of the success of our remedies will depend upon the time of administering them, the fidelity with which their operation is watched and promoted, and the care with which the comfort of the patient is preserved. Stillness, repose, a darkened but well ventilated chamber, all things, in a word, that mitigate his anguish, contribute to his restoration, and in the worst cases may be pronounced necessary to recovery.

---

*See Southwood Smith on Fever, for full details of the efficacy of this practice.

